# Soil Seed Bank of the Alpine Endemic Carnation, *Dianthus pavonius* Tausch (Piedmont, Italy), a Useful Model for the Study of Host–Pathogen Dynamics

**DOI:** 10.3390/plants13172432

**Published:** 2024-08-30

**Authors:** Valentina Carasso, Emily L. Bruns, Janis Antonovics, Michael E. Hood

**Affiliations:** 1 Centro Regionale Biodiversità Vegetale, Ente di Gestione delle Aree Protette delle Alpi Marittime, Via S. Anna, 34, 12013 Chiusa di Pesio, Italy; 2 Department of Biology, University of Maryland, 1204 Biology-Psychology Building, College Park, MD 20742-4415, USA; ebruns@umd.edu; 3 Department of Biology, University of Virginia, 485 McCormick Rd, Charlottesville, VA 22904, USA; ja8n@virginia.edu; 4 Department of Biology, Amherst College, 220 South Pleasant Street, Amherst, MA 01002, USA

**Keywords:** host–pathogen interactions, seed dormancy, *Microbotryum* spp., Maritime Alps, endemic species

## Abstract

Soil seedbanks are particularly important for the resiliency of species living in habitats threatened by climate change, such as alpine meadows. We investigated the germination rate and seedbank potential for the endemic species *Dianthus pavonius*, a carnation native to the Maritime Alps that is used as model system for disease in natural populations due to its frequent infections by a sterilizing anther-smut pathogen. We aimed to ascertain whether this species can create a persistent reserve of viable seeds in the soil which could impact coevolutionary dynamics. Over three years, we collected data from seeds sown in natural soil and analyzed their germination and viability. We found that *D. pavonius* seeds are not physiologically dormant and they are able to create a persistent soil seed bank that can store seeds in the soil for up to three years, but lower than the estimated plant lifespan. We conclude that while the seedbank may provide some demographic stability to the host population, its short duration is unlikely to strongly affect the host’s ability to respond to selection from disease. Our findings have implications for the conservation of this alpine species and for understanding the evolutionary dynamics between the host and its pathogen.

## 1. Introduction

Soil seed banks have multiple influences on plant communities and the dynamics of adaptation. Species persistence in natural habitats depends in part on the supply of seed trapped in the ground [1], and seeds that delay germination to subsequent growing seasons have been described as the “genetic memory” of a population [2,3]. Dormant seeds reflect the outcome of reproduction by plants growing in a past environment, and the level of dormancy can also be influenced by the temperatures experienced by the mother plant during seed development [4]. Dormant seeds may furthermore contain genotypes that have been lost from the actively growing population as a result of selection or genetic drift in small populations [5,6]. This preservation of alleles is particularly important for rare and endemic species that inhabit fragile or threatened environments, where the introduction of genetic variation by migration is limited [7,8,9], and where biotic and abiotic factors heavily affect the year-to-year survival of the above-ground plant community [10].

Although seed banks are often viewed as important reservoirs for genetic variation [3,11], extended seed dormancy may also slow the response to selection that is caused by short-term perturbations or shifts in ecological circumstances [6,12]. In a variable environment, the dormant seed pool can dampen cycles in genotype frequencies, and the seed bank may serve as an evolutionary filter that causes only the most severe years to have any lasting evolutionary impact [11]. This buffering aspect of seed dormancy implies that long-term environmental patterns are more likely to alter the genetic composition of a population through selection than are brief perturbations in the habitat [13].

The ability to respond to selection is of particular relevance to host–pathogen interactions, where the rapidity of coevolutionary dynamics is more extreme than most other biotic or abiotic forces [14,15]. Protection from disease, but also the costs of resistance in the absence of the pathogen, cause selection for or against resistance to frequently shift, especially because the extremes of infection prevalence can be cyclical or episodic [16,17]. Moreover, where there is genotype-specific compatibility between the host and pathogen (i.e., under ‘red queen’ or ‘arms race’ coevolutionary dynamics; [18]), selection acts against more common host genotypes, notably among the actively growing plants whose genotype frequencies may differ from dormant (or quiescent) seeds [19]. Such scenarios precisely represent the types of short-term shifts in directional or cyclical selection where seed banks may influence ongoing dynamics.

In the extreme conditions of high elevation or arctic environments, the role of seed banks can be more pronounced because lower soil temperatures and short growing seasons can lead to longer-lived seeds [2]. Alpine species are more often characterized as having “persistent” soil seed banks that remain viable for two or more years, as opposed to “transient” seed banks that only endure until the following growing season [20].

*Dianthus pavonius* Tausch is an iconic endemic plant native to the Western Maritime Alps. It is commonly found in alpine meadow habitats above 1500 m and can reach high densities (>100 plants per sq metre [21]). The plants are relatively long-lived, with an estimated average lifespan of 3.7–7.5 years [22]. Throughout its range, the plant is commonly infected with a pollinator-transmitted sterilizing ‘anther smut disease’ [23,24] caused by the Basidomycete fungus *Microbotryum dianthorum* (*sensu lato*). The current investigation follows the recent establishment of this wild alpine carnation as a study model for the ecology of infectious diseases in natural populations [25,26].

Here, we conducted a series of lab and field germination studies to understand the germination and seed bank of *D. pavonius* in the Maritime Alps of north-western Italy. We aimed to ascertain whether (1) the seeds of this species are physiologically dormant upon production, (2) if they are able to build a persistent soil seed bank lasting more than one year, and, (3) if so, to estimate the survival and germination of the dormant seed over subsequent years. This research is important for assessing the effects of the temporal lag between the foliar or floral pathogens and the viable seeds buried in the soil, and for a better understanding of the evolutionary dynamics between the host and pathogens in plant communities.

## 2. Results

### 2.1. Germination in the Lab

Seeds, which were freshly collected from the field in 2011 and lightly scarified, germinated readily on water agar medium, with a mean final cumulative germination percentage of 91 ± 0.01 (% ± SE, *n* = 4) after 24 days. The first germinations were observed after 3 days.

### 2.2. Germination of Seeds Sown at the Ground Level

**Figure 1:**
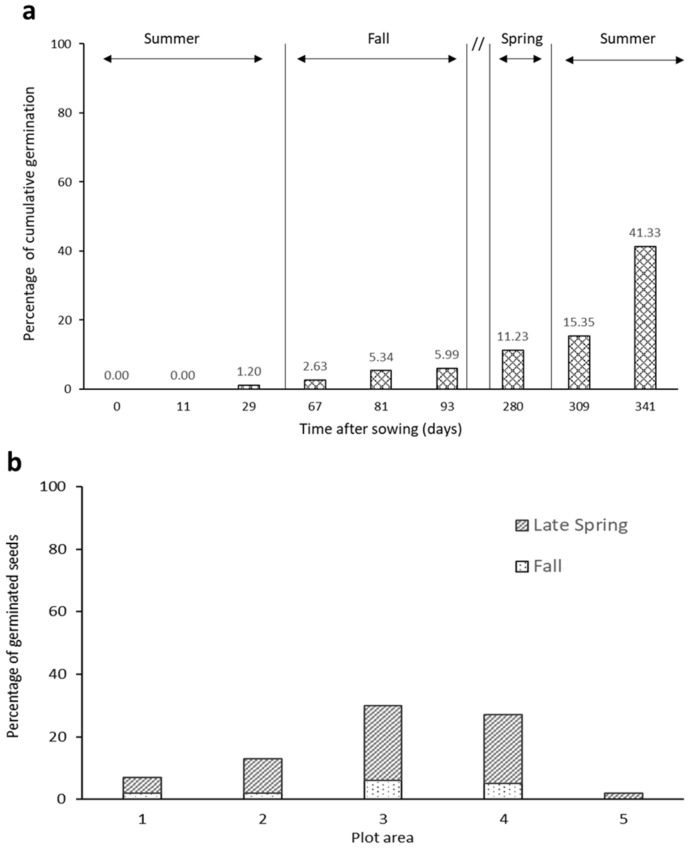
Germination at ground level. (**a**) Percentage of *Dianthus pavonius* seeds planted in August 2015 that had germinated during the fall of 2015 and late spring/summer of 2016. Data are summed over all five seed plots, and the cumulative percentage of seeds germinated is given in numbers above. (**b**) Percentage of seeds germinated in the fall vs late spring for each of the five plots consisting of 100 seeds.

Seeds of *Dianthus pavonius* are naturally dispersed in August. To determine when germination occurs, we planted 100 field-collected seeds in each of five different plots in August 2015 and tracked germination until July 2016. Total germinations along the whole duration of the experiment were low; just 83 out of 500 (17%) seeds germinated. Out of these 83 germinated seeds, 19 germinated that fall. The remaining 64 seeds that had germinated had done so during the following year in the late spring/early summer (Figure 1a). Within each of the five plots where seeds were planted, the observed germination was consistently higher after the winter than in the fall (Figure 1b).

To confirm that the fall germination rates of fresh seeds are low, and that there was not a chance event of poor conditions in 2016, we ran a repeat experiment in 2020. In this year, nearly all of the seeds sown in polyester bags in the soil (98%) were recovered 21 days after their placement in the alpine meadow. Among these seeds, the percentage of germinated seeds was just 6 ± 0.02 (% ± SE), while 90% were intact ungerminated seeds and the remaining 4% had damage from insects.

### 2.3. Long-Term Viability of Seeds Buried in the Soil

#### 2.3.1. Germination of Buried Seeds

We carried out 3-year seed burial experiments in the natural field and in nursery conditions (artificial plot).

**Figure 2:**
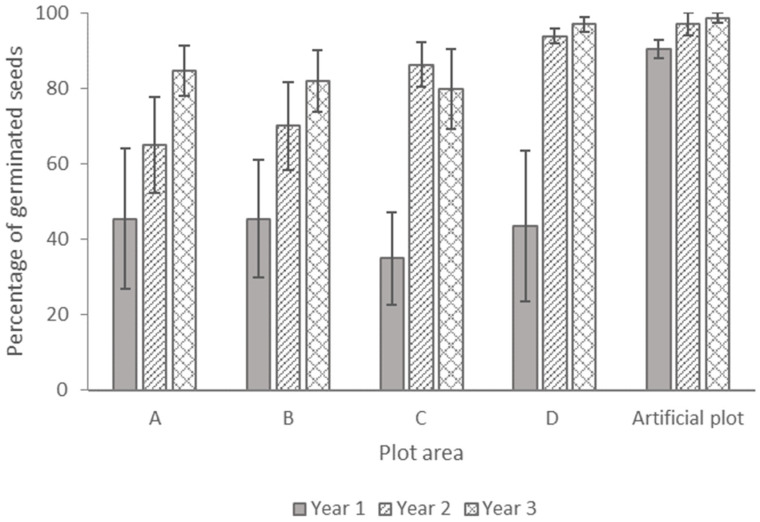
Percentage of cumulative germinated seeds (ratio between germinated seeds and total recovered seeds) of *Dianthus pavonius* when buried in the soil for 1 (bar in solid grey), 2 (diagonal lines), and 3 years (crosshatch) in different plots. Data are the means of four replicates (±SE).

In the nursery, the seeds were kept in a soil consisting of commercial ingredients and under regular watering conditions. After 1 year, 90% of seeds germinated, with then some further germination after years 2 and 3 (Figure 2).

In the field plots under natural conditions, in contrast, less than half of the seeds germinated after one year, with increases in the percentage germinated after 2 and 3 years (Figure 2). In the analysis of the four field plots, from which separate sets of seeds were retrieved from the soil each year, germinability varied among years (one-way ANOVA, F = 14.507, *p* < 0.001), and linear regression indicated that a higher percentage of germination was significantly predicted by the length of burial, but there was no significant effect from the experimental plot or the interaction term (Table 1). The average percentage of germinated seeds among the experimental plots after years 1, 2, and 3 were 41%, 79%, and 86%, respectively; posthoc test comparing overall germinations rates per year, 1 versus year 2, z = 10.376, corrected *p*-value = 0.0002, and year 2 versus year 3, z = 3.04, corrected *p*-value = 0.0048.

**Figure 3:**
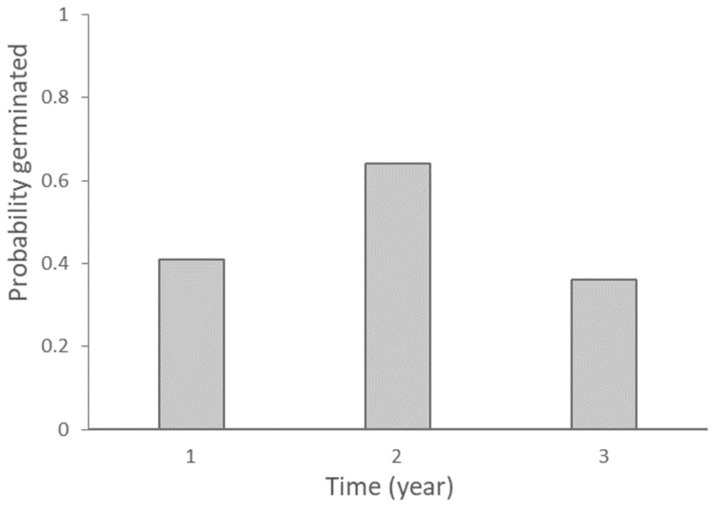
Probability of germination for ungerminated viable seeds of *Dianthus pavonius* per year under natural field conditions.

Using the estimated number of ungerminated seeds remaining in the samples after each successive year, the average probability of germination for ungerminated seeds per year was 0.41 at the end of the first year, increasing further in the second year to 0.64, but then reducing in the third year to 0.36 (Figure 3 and Supplementary File S1).

The probability of survival for ungerminated seeds per year was 1 minus the proportion of germinated seeds, and thus the average survival probability per year was 0.53. Therefore, the average time that a seed remains in the soil before germination, assuming a constant year to year survival, is 1.89 ± 0.09 years, or approximately 2 years.

#### 2.3.2. Seedlings from Buried Seeds

To verify the germination of the buried seeds, we examined the buried seeds for the presence of rooted seedlings or empty integuments in the seed packets.

**Figure 4:**
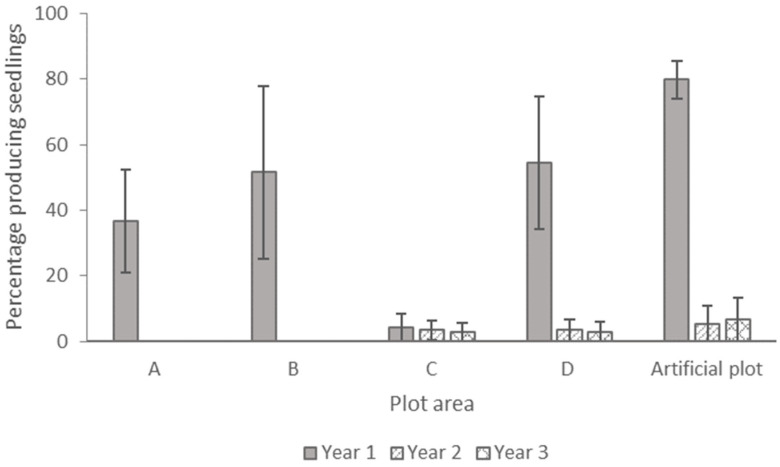
Percentage of *Dianthus pavonius* germinations with associated seedlings and empty integuments when buried in the soil for 1 (solid grey), 2 (diagonal lines), and 3 years (crosshatch) in different plots. Bars are the means of the four replicates (±SE).

After 1 year of burial (Figure 4), while the greatest amount of seedlings from germinated seeds was observed in the commercial soil in the nursery, a large variation among the replicate sample bags in the experimental field plots precluded statistical inference. In years 2 and 3, very few seedlings were observed (plots C and D and the artificial plot) or they were absent (plots A and B).

#### 2.3.3. Viability of Intact Buried Seeds

**Figure 5:**
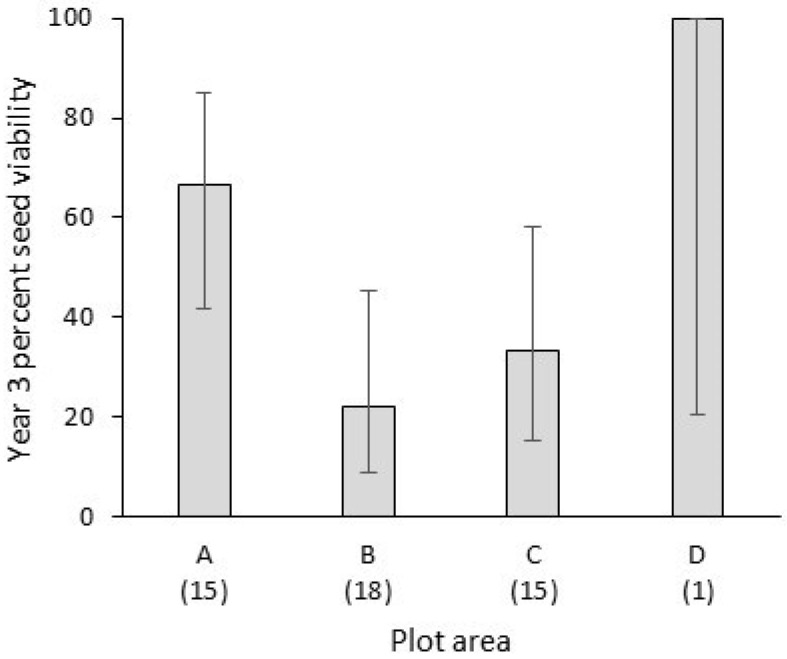
Percent viability of ungerminated seeds of *Dianthus pavonius* in year 3 of burial in experimental field soil plots. Parenthetical numbers below the x-axis indicate numbers of ungerminated seeds per plot (pooled among four replicate bags), and error bars indicate 95% confidence intervals.

A substantial percentage of seeds that were ungerminated in year 3 still maintained viability, providing the potential for germination to occur in a later year. Using the cut test with the TTC reagent, 21 of 50 ungerminated seeds (42%) across all of the experimental plots in year 3 were viable. Some viable seeds were found in each of the experimental plots even as the numbers of ungerminated seed and viability rate showed moderate non-significant variations among the plots (Figure 5).

## 3. Discussion

Our results show that seeds of this alpine carnation retain the ability to contribute to the population through germination of a proportion of seeds after more than two years residing in the soil. In addition, we found that although seeds are capable of germinating in the fall, the majority germinate in the late spring, indicating that while not strictly necessary, overwintering strongly improves germination.

In our work, fresh seeds of *Dianthus pavonius* are capable of germination immediately after the dispersal when scarified. While further studies are warranted on the eco-physiology of seed dormancy, the seed morphology has a flattened shape, and the integuments of fresh seeds are permeable to water and swell when wetted (Carasso, personal observations). Seeds are not light sensitive, as they can germinate both in the dark or in the light, as observed during the seed we rescued from the soil and additionally confirmed by Caser et al. [27]. The embryo, at the time of seed dispersal, is fully developed: this is in line with general features described for the *Dianthus* genus [28]. As seeds of this species, at the end of the summer, can germinate in temperatures around 20 °C, we assume that a cold requirement for germination is unlikely.

Also, a high rate of germinations (>91%) can be achieved with slight scarification of the seeds, the scarification likely causing a faster imbibition process (see [25]), although in other plants, wounds to the embryo from scarification can lead to germination [29]. These observations are consistent with other studies in which the same conditions were reported for optimal germination of other *Caryophyllaceae* genera like *Moehringia* and *Stellaria* [10,30,31], and particularly for several other Mediterranean *Dianthus* species where a lack of physiological dormancy was reported [32,33,34].

While there is a lack of physiological dormancy in *D. pavonius*, our field study provided strong evidence that, in the natural field plots, a substantial portion of buried seeds did not germinate after the first winter season and persisted in the soil as part of a seed bank. We observed a progressive increase over successive seasons in the total proportion of viable seeds that germinated, with the highest estimated probability of a viable seed germinating being in year 2 of the experiment. After three years, the soil still contained a small number of intact, viable seeds that could potentially contribute to seed germination in subsequent years. Thus, *D. pavonius* should be able to create a “persistent” seed bank, *sensu* Thompson and Grime [20], similarly with what has already been observed in other alpine species [35,36,37] or in other genera of the *Caryophyllaceae* family [5,38].

There are some other non-alpine *Dianthus* species where seed banks are “transient”. An example is given by the Mediterranean coastal species, *Dianthus morisianus* [27], for which after one year of burial very few seeds were retrieved intact. A similarly transient seed bank was also reported by Cerabolini et al. [39] for the temperate or lower elevation species, *D. monspessulanus*, *D. seguierii*, and *D. sylvestris*.

Even though internal physiological seed dormancy is an important mechanism promoting seed persistence in the soil, it is a poor predictor of seed bank persistence [40]. In fact, a phylogenetically controlled analysis showed that seed dormancy is not significantly related to seed bank density, with non-dormant species also being able to form dense seed banks [41]. The determinants of seed persistence in *D. pavonius* are likely to be aspects of the soil and climate that provide conditions permissive to germination, including soil texture and water availability. The small-scale environmental differences among the field plots then likely contributed to different patterns of year-to-year percentages of seed germination, and further studies could address how such variation may relate to environmental patterns of particular growing seasons or the progression of climate change.

### Consequences for the Host Population Biology and Host–Pathogen Dynamics

Our results provide the first insights into germination timing and seed bank properties of *D. pavonius*, with important consequences for disease dynamics. We found that the majority of seeds germinate in the late spring/early summer. This is important, because plants infected with the sterilizing anther-smut disease begin flowering in early July, very soon after germination, with spores produced by the fungus that can be aerially transmitted to seedlings [25]. Prior research has shown that young seedlings are significantly more susceptible to infection [25], and thus the late spring timing of germination ensures a new flush of highly susceptible host seedlings during just prior to the peak transmission period.

In addition, there are potential implications of a persistent seed bank for the evolutionary dynamics of host resistance to anther smut. Tellier and Brown [42] used a population genetic model to show that seedbanks could stabilize oscillating host–pathogen coevolutionary dynamics, with the stabilizing effect being stronger with more persistent seed banks.

In this study, we characterize *D. pavonius* as forming a short-term persistent seed bank *sensu* [43,44,45,46], remaining as seeds in the soil often for more than 1, but not often more than 3, years. For reference, the mortality rate of established flowering plants of *D. pavonius* is 0.12 [25], giving an estimated host lifespan as 8.3 years. The seed bank may provide some demographic stability to the host population, potentially affected by the plant vegetative lifespan [47] and species-specific or site-specific variation [48]. However, the short-duration seed bank in *D. pavonius* relative to its vegetative lifespan is unlikely to strongly affect the host’s ability to respond to selection from disease. Unfortunately, at the current state of our knowledge, we are not able to say conclusively whether a long-term persistent seed bank would or not promote resistance of the host plant. New in-depth empirical and theoretical investigations will need to be conducted, incorporating the observed seedbank properties reported here.

The outcomes from this work allow us to reflect on the threats that alpine (especially endemic) species face from changing climates and biotic antagonists. Soil seed banks could be an important source of short-term resiliency against particularly unfavourable years. Even the presence of a modest soil seed bank has important implications for land management and restoration. Although further investigations are necessary, the present work describes, for the first time, the presence of a short-term persistent soil seed bank in this alpine endemic species whose population is heavily affected by an infectious disease.

## 4. Materials and Methods

The research site was located in the Ligurian Alps (Piedmont, Italy) and precisely in high Valle Pesio (Cuneo Province). The main field site, named Pian del Lupo (44°18′98.4″ N, 7°69′15.0″ E), is part of the protected area of the Parco Naturale del Marguareis and consists of a large meadow at about 2000 m elevation characterized by a high geological and environmental diversity and a great floristic richness.

The vegetation is a seminatural pasture of the phytosociological alliance *Nardion strictae*. This pasture is partially degraded by nitrophilous species and the presence of some species characteristic of alliance *Triseto-polygonion bistortae*, showing past hay-making management [49].

The species used for this work is *Dianthus pavonius* Tausch., an alpine carnation that grows naturally in this area and is endemic to the Western Alps. Due to the brevity of the growing season, not all experiments could be run in the same year.

### 4.1. Check of Germination in the Lab

To assess seed germination, in 2011, 100 fresh seeds of *D. pavonius* were collected from the Pian del Lupo area. Because of the endemic status of the species, International Rules for Seed Testing were not applicable in this case. Therefore, available seeds were divided in four replicates, surface sterilized briefly in NaOCl 4.5% ethanol 96% with 1 drop of Tween 20 for 5 min followed by several rinses in sterile distilled water. The seeds were gently scarified (by nicking only the seed coat with a razor blade) placed on agar/water medium 0.8% *w*/*v* in 9 cm diameter Petri dishes, and incubated at constant 20 °C (photoperiod 12 h light/12 h dark); they were scored for germination twice a week for 24 days.

### 4.2. Check of Germination after Seed Sowing at the Ground Level

To determine whether germination occurred in the end of the summer/fall in which they were produced or following the winter, in August 2015 we set up a replicated seed germination study in Pian del Lupo meadow. Freshly collected *D. pavonius* seeds from mother plants in the same area were individually glued (ethyl 2-cyanoacrylate) to plastic toothpicks and put into 5 different replicate arrays located in the same field, with 100 seeds per array. Within an array, seeds were arranged at the ground level among the natural vegetation in a 10 × 10 grid with (5 cm) spacing between seeds. Seed arrays were checked for germination in September, October, and November before the snows, and again in May, June, and July the following year. For each plot, we calculated the percentages of new germinations in fall and in late spring out of the total number of seeds present at the beginning of the experiment.

A further experiment to assess the germination at the end of summer was set up at the time of natural dispersion in August 2020 in the Pian del Lupo meadow. Fresh seeds of *D. pavonius* were randomly collected from about thirty plants in the research area of Pian del Lupo. At this time, the seeds were still in the dry fruit, but detached from the placenta of the ovary. The seeds were extracted from the capsule, divided in 6 replicates of 25 seeds each, and put inside plastic “mesh cloth” bags with sieved natural soil. The replicate bags were randomly positioned in the same field, among the natural vegetation, near *D.s pavonius* adult plants, and directly exposed on the soil surface in order to reproduce the same conditions after the seed dispersion. Three weeks later, the bags were removed from the field and checked in the lab for the seed viability, germination, and seedling presence. The verification of these seed conditions was conducted under the stereoscope, dividing the seeds into the same categories described in the subsection of Section 4.3.

### 4.3. Check of Long-Term Viability of Seeds Buried in the Soil

To assess the potential for a multi-year seed bank, in August 2016, fresh seeds were randomly collected in the field of Pian del Lupo from about fifty plants. The seeds were moved to the lab, where they were cleaned from debris and counted. Underdeveloped, empty, or broken seeds were discharged. Prior to sowing in the field, seeds were stored in a drying room of the Piedmont Seed Bank (15–20% relative humidity and 15–20 °C temperature conditions) for 2 weeks.

The experimental design consisted of new square plots (1 × 1 m), topographically heterogeneous and randomly distributed within each of four areas within the Pian del Lupo meadow (Table 2), plus an “Artificial plot” positioned in the nursery of the Alpine Botanical Station of the Park located immediately adjacent to the field site. The artificial plot exposed the seeds to a commercial soil substrate rather than the natural soil of the alpine meadow and received supplementary watering during the summer seasons (twice a week for 15 min). At the beginning of September 2016, 12 polyester confetti bags with 25 seeds each of *D. pavonius*, together with previously sieved natural soil, were placed at the corners of the square area of each plot and buried about 3 cm under the soil surface and among the natural vegetation. The distance between the mother plants and the sowing area did not exceed 100 m. As seed rain and germination interact only with the superficial seed bank [2], we considered this layer (0–3 cm deep) to be the active component of the soil bank. For the artificial plot located in the nursery, the bags were filled with a prepared soil mix of commercial ingredients (Table 2) and the seed, and then the bags were buried in a plastic tray.

The principal features of the natural experimental field plots in the study area and the control plot are described in Table 2.

#### Recovering the Seed Bags Buried in the Soil

After the seed burial in August 2016, in June of each of the following three years (2017–2019) a separate set of 4 bags (each originally with 25 seeds inside) was retrieved from each of the experimental and artificial plots. The duration of the seeds burial in the soil corresponds to 9 months for those of year 1 and an additional 12 or 24 subsequent months for those of years 2 and 3. The seed bags buried in the field were found using a metal detector and with iron wire previously fixed in the middle and at the corners of the plots. Each year, the retrieved bags were moved to the lab and analyzed in order to check the seed viability, germination, or decay. The material in the bags was scored under a stereoscope and divided in three categories:

1. Germinated seed;
2. Seedling associated with a seed coat;
3. Ungerminated seed (subcategorized as viable or inviable).

Seeds were scored as ‘germinated’ (category 1) if they were intact and empty integuments with a micropylar hole were observed, or as ‘seedling’ if visible cotyledons were found to be associated with a given seed coat (category 2). ‘Ungerminated seed’ (category 3) included intact firm, intact non-firm, and decayed seeds. Ungerminated seeds that were firm were checked for viability using a ‘cut test’ and the reaction of the seed contents with the chemical TTC (2,3,5-triphenil-tetrazolium-cloride). Cut seeds were put in a solution of 1% *w*/*v* TTC and incubated overnight at 35 °C in the dark. The seeds were then rinsed several times with distilled water and observed with the stereoscope for the colour indicator of viability, where red indicates viable tissue and white indicates inviable tissue. In addition, ungerminated seeds that were not firm or that were decayed were considered to be non-viable.

### 4.4. Data Analysis

The final germination percentage in a given year was obtained as the number of germinated seeds over the number recovered from the buried samples. The percentage of germinations that produced seedlings (category 2) was obtained as the number of observed seedlings over the total number of germinated seeds each year. The percentage of seeds that were viable among the ungerminated seed was obtained as the number showing a positive reaction (full red tissues) with the chemical TTC over the total number of ungerminated seeds.

Whether the seed germination rate differed among years was checked using a one-way analysis of variance (ANOVA). Excluding the artificial plot, arcsine square-root transformed proportional germination data were used for a linear regression to assess if the germination proportions were significantly predicted using years as a variable with ordinal values, using differences among experimental field plots, and using the interaction of years and plots. Analyses were conducted in SPSS version 27. The percentage of seeds having germinated per year was calculated using the mean of the final germination percentage obtained for the four experimental field plots in each year. A posthoc text of germination differences between years 1 and 2 and between years 2 and 3 was performed using z-tests, with *p*-values corrected for multiple comparisons using the Bonferroni method. The probability of germination for remaining ungerminated seeds per year was calculated as the mean number germinated in that year minus the sum of the germinated seeds in prior years, over 25 minus the sum of the germinated seeds in prior years, i.e., where Y is the mean number of germinated seed denoted in year *n*, for year 2 equals (Y_n_ − Y_n−1_)/(25 − Y_n−1_).

## Figures and Tables

**Table 1:** Analysis of seed germination of *Dianthus pavonius* in the seed buried experiment among years and experimental field plots, including a linear effect of year, plot, and their interaction (data arcsin square root transformed).

Test of Model Effects—Type III			
Source	Wald Chi-Square	df	*p*-Value
(Intercept)	45.514	1	<0.0001
Year	60.362	1	<0.0001
Plot	0.370	3	0.946
Plot × year	4.054	3	0.256

**Table 2:** Description of test sites where natural and artificial plots were placed.

Plot	Altitude (m a.s.l.)	Plot Location	Plot Morphology	Dominant Species
A	1969 m	44°11′27″ N 7°41′24″ E	Mountain slope	*Vaccinium myrtillus*, *Nardus stricta*, *Dianthus pavonius*
B	1962 m	44°11′27″ N 7°41′23″ E	Mountain slope	*Juniperus communis*, *Nardus stricta*
C	1963 m	44°11′23″ N 7°41′17″ E	Flat alpine grassland	*Nardus stricta*, *Euphrasia minima*
D	1968 m	44°11′24″ N 7°41′17″ E	Hilltop on debris	*Rhododendron ferrugineum*, *Euphrasia minima*, *Nardus stricta*
Artificial plot	1961 m	44°11′23″ N 7°41′18″ E	Plant nursery in the Alpine Botanical Station	No vegetation

## Data Availability

Data is contained within the article and in Supplementary Material.

## Supplementary Materials

The following supporting information can be downloaded at: https://www.mdpi.com/article/10.3390/plants13172432/s1, Figure S1: Probability of germination in the four plots for ungerminated viable seeds of *Dianthus pavonius* per year and under natural field conditions; File S1: Soil seed bank of the alpine endemic carnation, *Dianthus pavonius* Tausch (Piedmont, Italy), a useful model for the study of host-pathogen dynamics.
