# Supplementary material for: Soil Seed Bank of the Alpine Endemic Carnation, Dianthus pavonius Tausch (Piedmont, Italy), a Useful Model for the Study of Host–Pathogen Dynamics
Source: Plants (Basel). 2024 Aug 30;13(17):2432. doi: 10.3390/plants13172432 (PMC11397626; doi:10.3390/plants13172432)
Supplement: Supplementary file 1 [file plants-13-02432-s001.zip › Figure S1.pptx]

## Slide 1
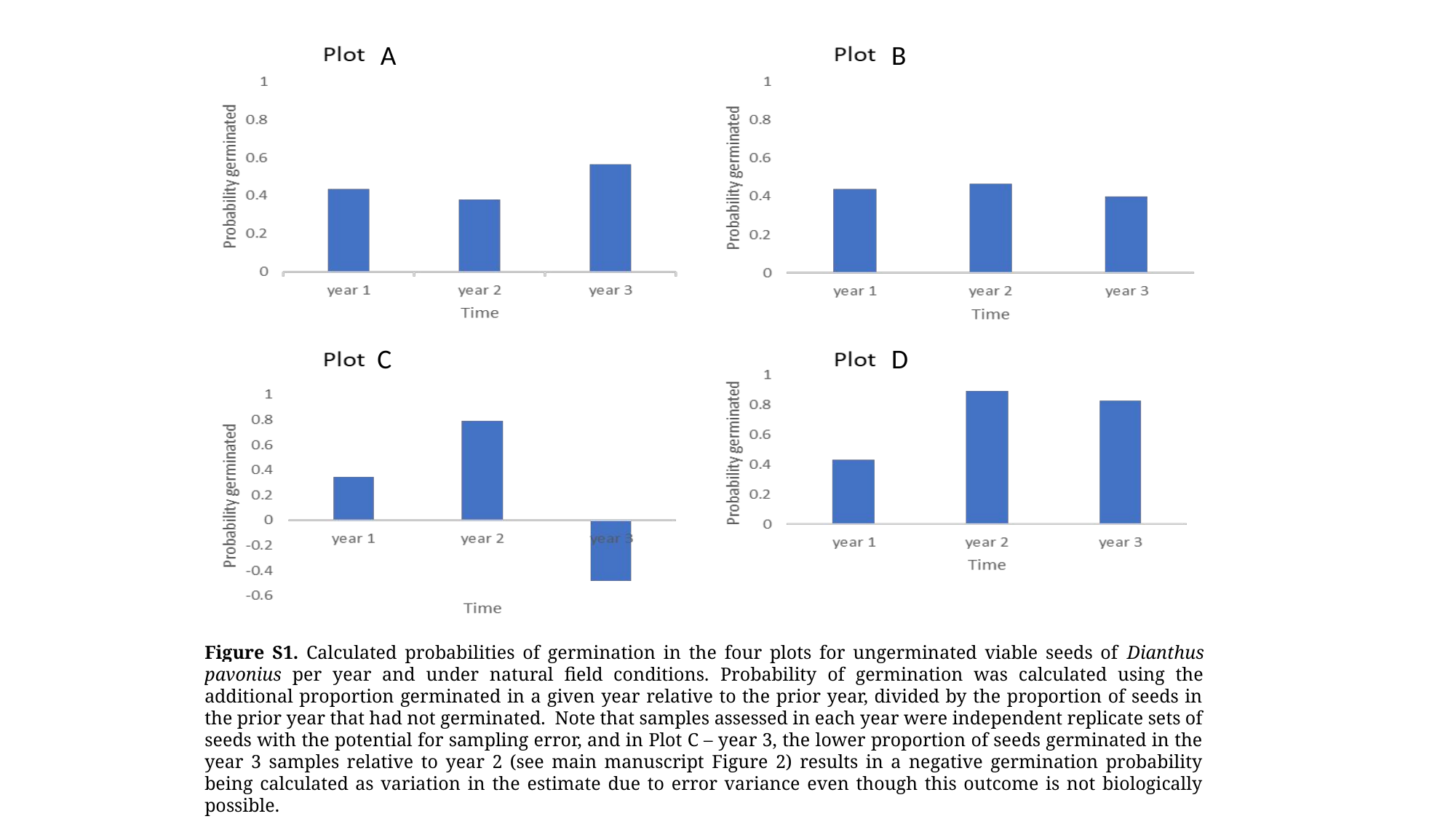

A
B
C
D
Figure S1. Calculated probabilities of germination in the four plots for ungerminated viable seeds of Dianthus pavonius per year and under natural field conditions. Probability of germination was calculated using the additional proportion germinated in a given year relative to the prior year, divided by the proportion of seeds in the prior year that had not germinated. Note that samples assessed in each year were independent replicate sets of seeds with the potential for sampling error, and in Plot C – year 3, the lower proportion of seeds germinated in the year 3 samples relative to year 2 (see main manuscript Figure 2) results in a negative germination probability being calculated as variation in the estimate due to error variance even though this outcome is not biologically possible.
